# A Low-Cost, Stand-Alone Sensory Platform for Monitoring Extreme Solar Overirradiance Events

**DOI:** 10.3390/s18082685

**Published:** 2018-08-15

**Authors:** Otavio Andre Chase, Mailson Borges Teles, Marinaldo de Jesus dos Santos Rodrigues, José Felipe Souza de Almeida, Wilson Negrão Macêdo, Carlos Tavares da Costa Junior

**Affiliations:** 1 Cyberspatial Institute (ICIBE), Amazonian Federal Rural University (UFRA), Av. Presidente Tancredo Neves, 2501–Montese, Belém, PA 66077-830, Brazil; felipe.almeida@ufra.edu.br; 2 Group of Studies and Development in Alternative Energies (GEDAE), Institute of Technology (ITEC), Federal University of Pará (UFPA), Rua Augusto Corrêa, 01–Guamá, Belém, PA 66075-110, Brazil; mbteles@ufpa.br (M.B.T.); marinaldo.rodrigues@abaetetuba.ufpa.br (M.d.J.d.S.R.); wnmacedo@ufpa.br (W.N.M); cartav.ufpa@gmail.com (C.T.d.C.J.)

**Keywords:** environmental monitoring, low-cost systems, sensory platform, solar irradiance

## Abstract

In this paper, we present a low-cost, stand-alone sensory platform developed for in situ monitoring of environmental parameters, for use in the Amazon region in the north of Brazil. The mission of the platform is to perform monitoring and identification of overirradiance (solar irradiance > 1000 W/m^2^) and extreme overirradiance events (solar irradiance > 1300 W/m^2^) using a photovoltaic based irradiance sensor. The sensory platform was built using the ESP8266 microcontroller, an open embedded computer capable of Wi-Fi communication using the IEEE 802.11 standard, and small photovoltaic modules, air temperature, atmospheric pressure, voltage, and current sensors, enabling the development of a low-cost system (€70/R$350.00 BRL). Calibration and tests were conducted at the Federal University of Pará (UFPA), Belém campus, Pará, where the platform measured an extreme overirradiance of 1321 W/m^2^ at a low-latitude (1 °S) and low altitude (7 m above sea level).

## 1. Introduction

Photovoltaic devices are typically designed considering terrestrial solar irradiance (*G_SI_*), that is the solar energy flux incident on a completely flat 1 m^2^ area of ground, which is usually ~1000 W/m^2^ (typically at 12:00). This value is a function of the interaction of the extra-terrestrial solar irradiance (*G_SC_*, mean value is 1361.1 W/m^2^) with the air mass (AM) in the Earth’s atmosphere [1].

Contrary to perception, solar irradiance peaks happen in partially cloudy weather conditions [2]. While clouds are generally the main attenuator of solar irradiance, in certain conditions, they amplify *G_SI_* to values above 1000 W/m^2^, and even *G_SC_*, causing overirradiance (>1000 W/m^2^) and extreme overirradiance events (>1300 W/m^2^) [3,4]. The occurrence of these events is a result of the cloud enhancement effect, where the combination of cloud density and coverage at values between 50% and 90% of the sky, along with zones of clear-sky, is able to increase solar irradiance [5]. Overirradiance events have been shown to last from seconds to minutes, depending on the velocity of cloud motion [6]. 

**Figure 1:**
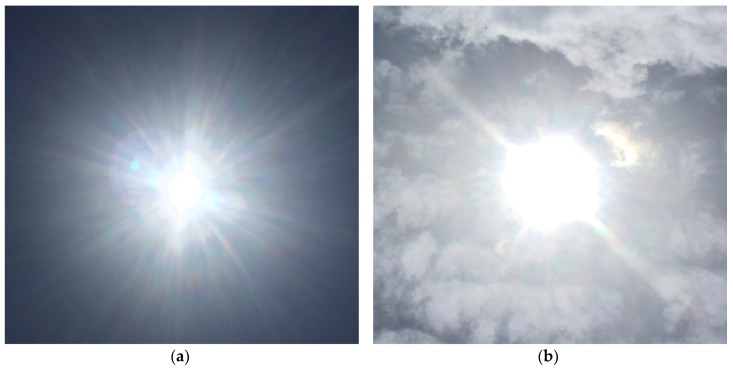
Appearance of solar irradiance in (**a**) clear sky, and (**b**) with cumulus clouds surrounding the solar disk. These images do not necessarily represent the moments of maximum irradiance.

The cloud enhancement effect is typically observed as the reflection of light over the edge of broken cumulus clouds around the solar disk, or the omnidirectional scattering of light when solar irradiance penetrates the base of the thin cumulus clouds [4,7]. Both of these occurrences can be explained by Mie scattering phenomena [8]. Figure 1 illustrates the difference between the appearance of solar irradiance in a clear sky (Figure 1a) and on a partly cloudy day (Figure 1b). Table 1 summarizes locations around the world where overirradiance events have been registered.

Overirradiance can cause arcing in photovoltaic modules, leading to fires and loss of property [6]. This increase in irradiance also increases the current injected into an electrical system, which can trigger the protection devices of photovoltaic systems, and even damage inverters, if the irradiance exceeds 1200 W/m^2^ [4]. In spite of tolerances of 20–25% included by some manufacturers of photovoltaic systems, a considerable amount of solar energy is still neglected because of its intensity [7,10]. Recent studies show that these tolerances do not consider the effect of extreme overirradiance events on sub-cable sizing and protection devices for photovoltaic systems, particularly with respect to overcurrent protection [4,10,11]. Luoma and Burger [4] quantify energy losses of up to 6.5% of the monthly energy produced by a PV array and inverter system (San Diego, CA, USA), due to inverter saturation by overirradiance events.

Based on the above considerations, overirradiance events should be monitored to ensure the stability of solar power generation. These results were obtained using a range of instruments, such as pyranometers, photovoltaic cells or modules, data loggers, and energy analyzers for data acquisition, with significant differences in their sensor response time. These range from 10^−5^ s to 18 s, as measured by the time required for the sensor to reach 95% of its final value following a step input. The temporal measurement resolutions of these instruments also differ significantly, varying between 10^−5^ s and 300 s. Sensors with shorter response times and measurement resolutions are able to record overirradiance events better, since these only last between a few sub-seconds or several seconds [3,4,7,8,9,12,13]. Hence, a sensor such as the thermopile pyranometer, which has a slow response time of 18 s on average, is not recommended for monitoring overirradiance [6].

In spite of the information in Table 1, many of these studies either do not specify in detail the sensors and data acquisition and processing devices used in research, or employ expensive instruments, making it difficult to implement these monitoring solutions in photovoltaic installations with few resources, or in developing countries like Brazil. 

In this work, we present a low-cost sensory system for in situ monitoring of overirradiance events. We employed open hardware in implementing these modifications, as their low cost [14,15,16], in comparison to commercial hardware, makes the development of solutions for monitoring overirradiance events in places with limited resources possible. The sensory platform measures, analyses, and submits solar irradiance, air temperature, and atmospheric pressure parameters to the Internet of Things (IoT), a network of physical objects and devices embedded with electronics, software, network connectivity, and sensors that facilitates the collection and exchange of data [17]. We used monocrystalline silicon (mono *c-Si*) photovoltaic modules for irradiance sensing because of their efficiency, as they are able to generate more energy than polycrystalline silicon (poly *c-Si*) photovoltaic module with a similar area, even in cloudy weather, or with low light [18]. The distinctive features of the designed system can thus be summarized as follows. We created an IoT-enabled sensory platform with a low-cost architecture based on open hardware that is powered by an embedded photovoltaic system with a mono *c-Si* PV module. The platform employs a separate PV-based mono *c-Si* sensor for solar irradiance monitoring and was tested in the Amazonian region at the Group of Studies and Development in Alternative Energies, Federal University of Pará (GEDAE/UFPA). This work is the first considering the monitoring of overirradiance events at low latitude (1 °S) and low altitude (7 m above sea level (a.s.l.)).

The rest of this paper is structured as follows. Section 2 illustrates the architecture of the sensory platform and explains the scheduling of tasks for monitoring overirradiance events. The test site and experimental configuration are discussed in Section 3. Section 4 details the results of the operation of sensory platform. Finally, conclusions and ideas for future work are presented in Section 5.

## 2. Sensory Platform Architecture

**Figure 2:**
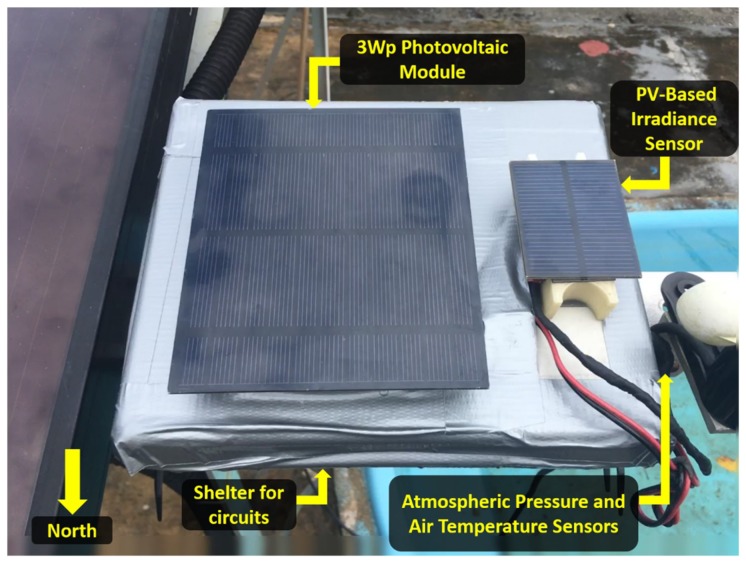
The sensory platform at the test site at the GEDAE/UFPA, tilted 10° to the north.

The sensory platform presented in Figure 2 includes sensors to monitor three parameters, solar irradiance, air temperature and atmospheric pressure. The electronic components of the system were placed in a plastic box with an IP67 rating, making them dust and water resistant [18]. Individual components are considered in further detail below. 

**Figure 3:**
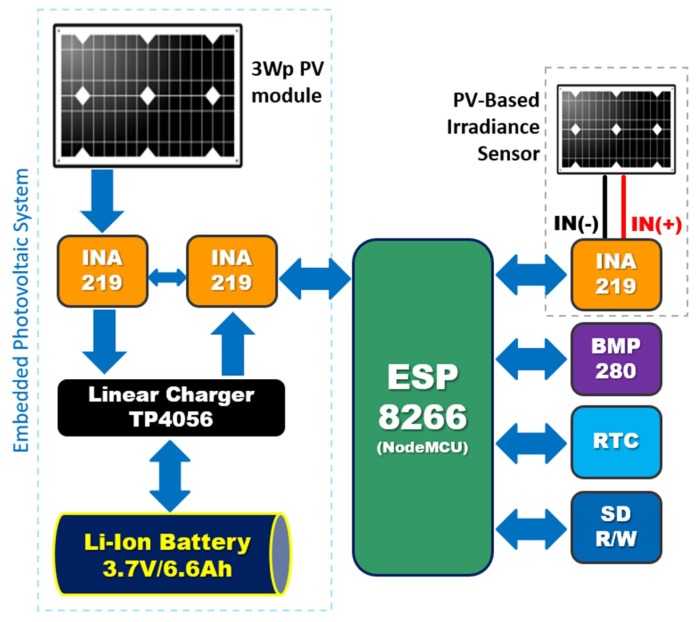
Block diagram of the device platform.

The platform consists of a BMP280 sensor for air temperature and atmospheric pressure, three INA219 current shunt and power sensors, a DS3231 real time clock (RTC), a TP4056 DC linear charger for Li-ion batteries, and two mono *c-Si* PV modules. Data communication is performed using an ESP8266-12E embedded computer, located on a NodeMCU board (v1.0). This component is a low-cost IoT enabler, as it is both a microcontroller (with a 32-bit processor) and Wi-Fi transceiver, with a complete TCP/IP (transfer control protocol/internet protocol) stack based on 2.4-GHz IEEE 802.11 (b/g/n). The current consumption of the chip ranges from 10 µA in low-power mode (deep sleep) to 200 mA (operating at maximum transmission capacity) [19]. A block diagram of how these components are connected is presented in Figure 3.

The embedded photovoltaic system generates electric power for the platform, using a 3-Wp mono *c-Si* PV module, manufactured by SEEED, with a short circuit current (I_SC_) of 540 mA, an open-circuit voltage (V_OC_) of 6.2 V_DC_, and 17% efficiency [20]. This energy is stored in a Li-ion battery bank equivalent to a single 3.7 V_DC_/6.6 Ah (24.42 Wh) source. The battery bank comprises three 2200-mAh ICR18650 batteries in parallel [21]. The TP4056 DC linear charger (current consumption of 0.5 mA) balances the load between the photovoltaic module and battery, protecting it and the circuit from overloading [22]. The NodeMCU board includes a voltage regulator that converts the 3.7 V_DC_ signal generated by the TP4056, to the 3.3 V_DC_ required by the ESP8266-12E, which subsequently powers the remaining devices. 

The need for current and power sensors can be explained by considering the typical operation of the system. The platform registers the instantaneous solar irradiance, air temperature and air pressure every second, between 06:00 and 17:45 each day (49% of the daily cycle), with a power consumption of 170 mW. It subsequently enters low-power mode (deep sleep) between 18:01 and 05:59 (50% of the daily cycle), in which the consumption is 25 mW. At the end of a daily monitoring cycle, between 17:45 and 18:00, the Wi-Fi modem is activated for data transmission to the IoT broker maintained by ThingSpeak, a process which consumes 300 mW of power. This data transmission constitutes 1% of the daily cycle. Using the average weighted power of the different time periods, the daily energy consumption can be calculated as,

(1) $$(Time\times weight{)}_{1}\times Power_{1}+(Time\times weight{)}_{2}\times Power_{2}+\cdots +(Time\times weight{)}_{n}\times Power_{n}\;∴(24\;h\times 0.49)\times 170\;\mathrm{mW}+(24\;h\times 0.50)\times 25\;\mathrm{mW}+(24\;h\times 0.01)\times 300\;\mathrm{mW}=2371.2\;\mathrm{mWh},$$

from the above calculation, without the embedded photovoltaic system, the platform would only be functional for approximately 10 days (24.42 Wh/2.37 Wh). To facilitate the optimal operation of this system, the platform includes power monitors, in the form of the INA219 chip, which draws a current of 1 mA. Integrated into this device are a 0.1 Ω shunt resistor (2 W), accurate to 1%, for measuring power, a 12-bit A/D converter, and I^2^C (Integrated-Integrated Circuit) communication. This sensor has a detection range of ±3.2 A with a resolution of 0.8 mA, and a response time of 68 milliseconds. Measurements with this chip have an accuracy of ± 0.5% [23]. Two of these devices acquire data for load balancing: The first measures the power generated by the photovoltaic module, and the second measures the energy consumption of the entire circuit.

In addition to the information relating to the energy consumption of the circuit, data collected by the sensors is stored on a 2-GB secure digital (SD) memory card, which consumes a current of 0.18 mA. Communication between ESP8266 with sensors and the SD card is performed through a serial peripheral interface (SPI) bus [24]. The DS3231 RTC generates the time reference for the platform, and communicates this through the I^2^C bus, a process which consumes 3 mA of current [25]. 

Air temperature and atmospheric pressure measurements are produced by the BMP280 sensor, manufactured by Bosch. This device has an internal 16-bit A/D converter, and features I^2^C communication, while consuming a current of 24.8 µA. Atmospheric pressure can be measured in a range from 300 to 1100 hPa (±0.12 hPa), while temperature can be measured between −40 to 85 °C (±1.0 °C) [26]. We used a 0.5-Wp mono *c-Si* PV module, manufactured by SEEED, for monitoring irradiance, based on variations to its short circuit current (*I_SC_*). In standard test conditions (STC), the module exhibits a short circuit current (*I_SC_*) of 100 mA, and an open-circuit voltage (V_OC_) of 5.5 V_DC_, with a spectral range of 350 nm to 1150 nm, a 10^−5^ s response time, and an efficiency of 17% [27]. To read the value of *I_SC_*, the positive and negative (GND) terminals of the photovoltaic module are connected to the *IN*(+) and *IN*(−) pins of an INA219 chip, respectively (Figure 3). The conversion of I_SC_ to an irradiance in W/m^2^ is performed as below [28]:

(2) $$Gi_{PV}=\frac{Read(I_{SC})\times Gi_{ref}}{I_{SC\;ref}},$$

where *Gi_PV_* is the solar irradiance on the surface of the photovoltaic module W/m^2^, *Read*(I*_SC_*) is the short-circuit current measured by the INA219 chip, *Gi_ref_* is the reference value for terrestrial solar irradiance (*G_SI_*), and *I_SCref_* is the maximum possible short-circuit current that can be generated by the PV module in STC. 

Calibration of the PV-based irradiance sensor (PV module + INA219 chip) was performed in real operating conditions by comparing the sensor’s response to that of the ISET01796 mono *c-Si* PV-based irradiance sensor [29], with signals read by the Fluke 435 energy analyzer [30]. To ensure that this calibration is valid, we calculated the coefficient of determination (R^2^ = 0.9976) and correlation (R = +0.9987) between irradiance measurements obtained by both sensors on the same day. Hence, the calculation required to correct the magnitude error, ensuring that the results of measurements are similar to those obtained by the previously-validated ISET0176 sensor, is given as,

(3) $$Gi_{m}=1.0295\times Gi_{PV}-8.4574,$$

where *Gi_m_* is the calibrated solar irradiance in W/m^2^. The temporal resolution of the instantaneous measurements is 1 s, with a total of approximately 40,000 measurements performed daily (3.3 MB per day). By combining (3) and (4), the equation relating *I_SC_* to *Gi_m_* can be obtained as below:

(4) $$Gi_{m}=1.0295\times (\frac{Read(I_{SC})\times 1000}{0.100})-8.4574,$$

**Figure 4:**
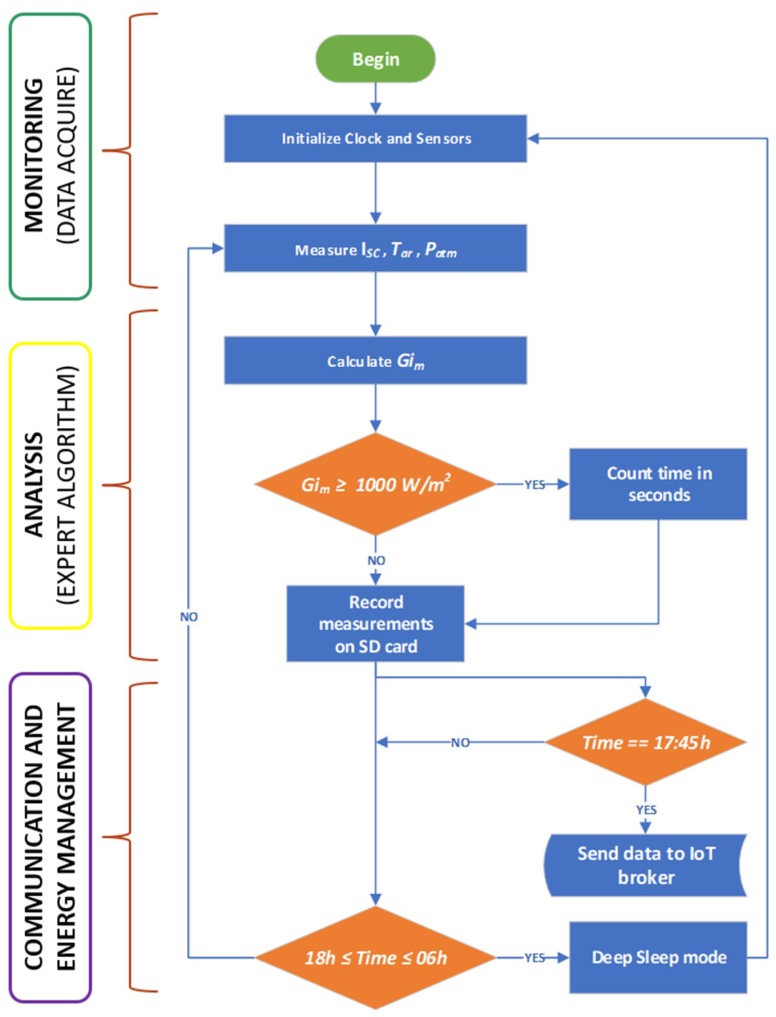
Flowchart illustrating the operation of the sensory platform.

This equation is implemented in an expert algorithm in the microcontroller, which uses the results of calculations to identify irradiance values above 1000 W/m^2^. The algorithm subsequently classifies these as overirradiance events and computes their duration. A flowchart illustrating data processing in the platform is shown in Figure 4.

**Figure 5:**
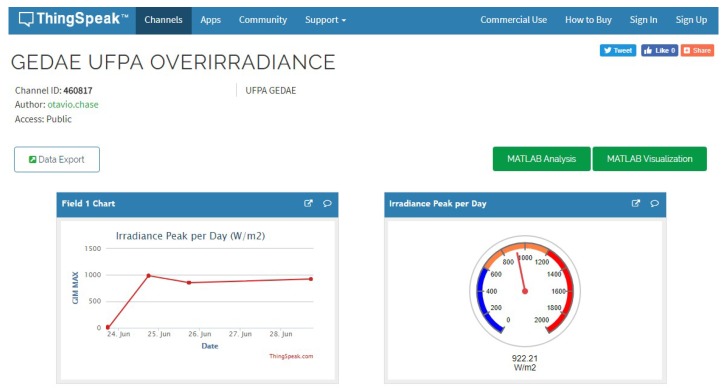
Illustration of the ThingSpeak interface (Available at: https://thingspeak.com/channels/460817).

The processed irradiance data can be viewed on the IoT broker (server) (illustrated in Figure 5). In this work, we use the free plan of ThingSpeak, a tool with a focus on IoT services developed by Mathworks [19], as the IoT broker. In addition to data reception and storage, this tool allows the development of data plotting scripts that process and generate charts online, from the Matlab program. The broker centralizes communications, decoupling the interaction between notifying parties, which is not possible in other client/server communication models. The IoT provides a full infrastructure for dissemination of sensors on location (in situ) or remotely (ex situ). The platform is connected to the GEDAE/UFPA Wi-Fi network. Finally, Table 2 presents a budget for the components in the platform, the total cost of which is €69.31.

When compared to the average cost of thermopile/photodiode pyranometers (€350), commercial dataloggers (€500), and energy analyzers (€4000), the designed platform can be considered to be a low-cost irradiance monitoring solution. In addition, the system is scalable, as new sensors and algorithms can be added as new demands arise, something that cannot be done intuitively in proprietary systems.

## 3. Test Site

**Figure 6:**
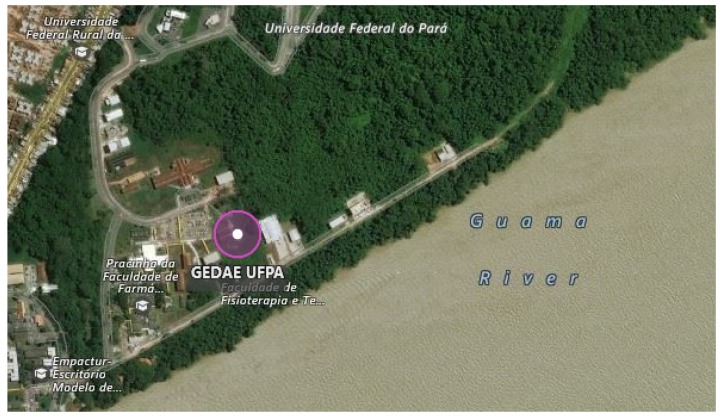
Location of GEDAE/UFPA at 1 °S and 7 m a.s.l.

The platform was tested at GEDAE on the UFPA campus (Figure 6), near the city of Belém in Pará state, on south latitude 1°28′13.677′’ S and west longitude 48°26′44.597′’ W, at an elevation of 7 m.

The region has a tropical rainforest climate, designated as *Af* under the *Köppen-Geiger* climate classification system, influenced directly by the presence of the Amazon rainforest [31]. The average annual air temperature in this region is 26.5 °C, with average peak sun hours of (HSP) 5.05 kWh/m^2^ per day, corresponding to 2000 h of sunshine/year [32]. The brightest period of the year lasts 2.9 months, from 8 August to 5 November, with an average daily radiation of 6.3 kWh in this period. The darkest period of the year lasts 4.4 months, from 14 January to 16 May, with an average daily radiation of 4.6 kWh [33]. 

The percentage of the sky covered by clouds varies significantly over the course of the year, according to the season. The brighter part of the year in Belém begins around 9 June and lasts for 4.5 months, ending around 27 October. The cloudier part of the year begins around 27 October and lasts for 7.4 months, ending around 9 June [33].

Overirradiance measurements were conducted for 4 days, between 10 May 2018 and 13 May 2018. The platform was fixed horizontally, with an inclination of 10° towards geographic North.

## 4. Results

**Figure 7:**
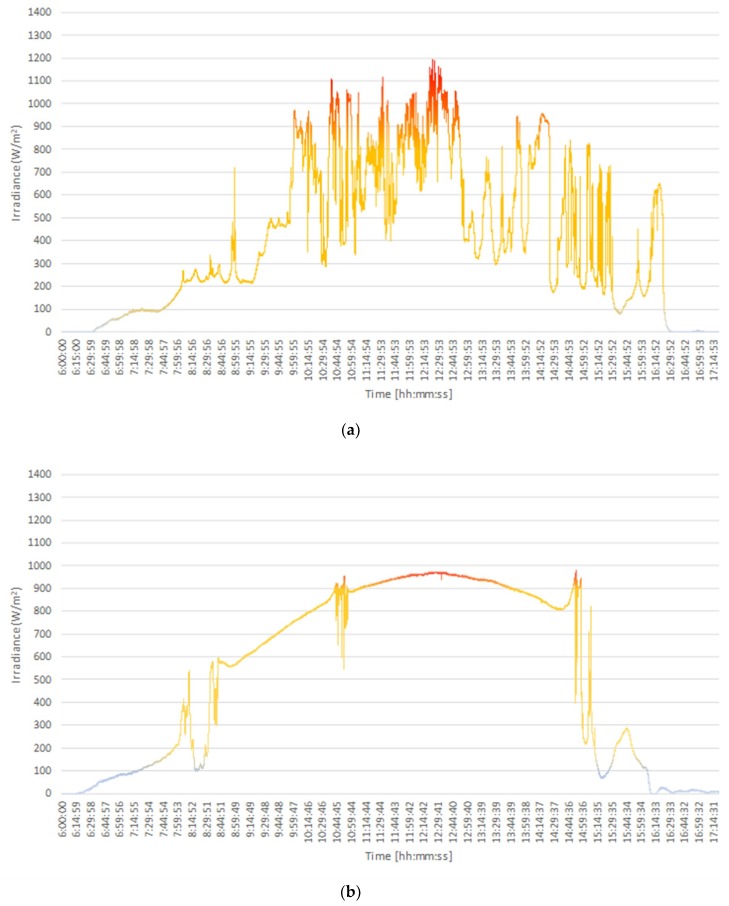
Irradiance measurements collected on (**a**) 10/05/2018 (maximum irradiance of 1193 W/m^2^ at 12:23:54) and (**b**) 11/05/2018 (maximum irradiance of 979 W/m^2^ at 14:52:43).

Figure 7 indicates the results of irradiance measurements performed on 10 and 11 May. The highest irradiance registered on 10 May was 1193 W/m^2^ (±6 W/m^2^), measured at 12:23:54 (Figure 7a), with an air temperature of 40.5 °C and an atmospheric pressure of 1012 hPa. This day was partially cloudy, with rain beginning from 13:00. On this day, overirradiance events (values above 1000 W/m^2^) constituted 4.5% of all measurements. On 11 May (Figure 7b), the highest irradiance, measured at 14:52:43 with an air temperature of 48.1 °C at an atmospheric pressure of 1009 hPa, was 979 W/m^2^ (±4.9 W/m^2^). The day was clear until 15:00, when it began to rain. No overirradiance events were measured on this day.

**Figure 8:**
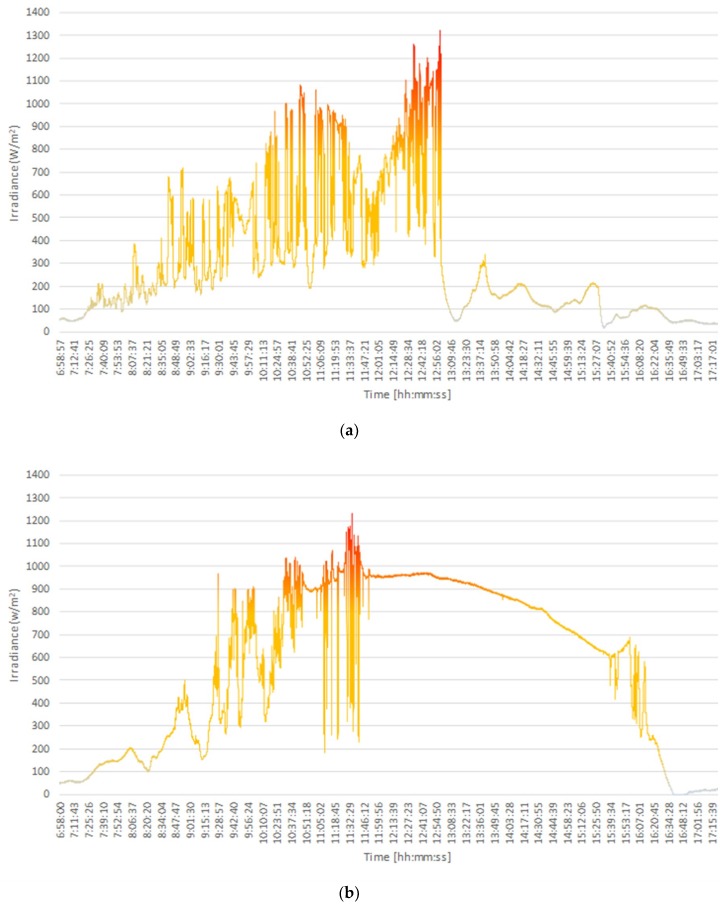
Irradiance measurements collected on (**a**) 12/05/2018, where an extreme overirradiance of 1321 W/m^2^ was observed at 12:59:43, and (**b**) 13/05/2018 (maximum irradiance of 1232 W/m^2^ at 11:35:06).

Figure 8 indicates the results of measurements performed on 12 and 13 May. An irradiance of 1321 W/m^2^ (±6.6 W/m^2^) was measured at 12:59:43 on May 12, with an air temperature of 44.9 °C and an atmospheric pressure of 1011 hPa (Figure 8a). To the best of our knowledge, this is the highest irradiance recorded at a latitude 1 °S and an altitude of 7 m a.s.l. On this day overirradiance events constituted 3.1% of all measurements. The highest irradiance on May 13 (Figure 8b), measured at 11:35:06 with an air temperature of 41.5 °C at an atmospheric pressure of 1011 hPa, was 1232 W/m^2^ (±6.16 W/m^2^). This day was partly cloudy until 11:50, after which, it was clear. On this day overirradiance events constituted 2.1% of all measurements.

**Figure 9:**
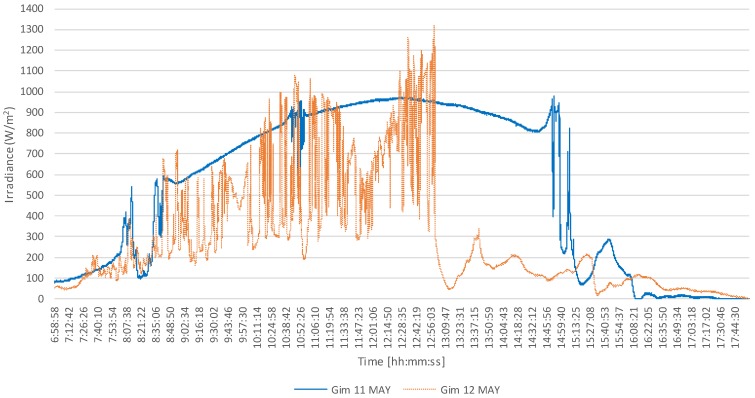
Comparison between the instantaneous irradiances recorded on 11 and 12 May 2018.

Figure 9 presents a comparison between the measurements obtained on 11 and 12 May, which highlights the effect of cloud cover on the magnitude of irradiance. As previously stated, the maximum irradiance recorded on 11 May, which was a clear day, was 979 W/m^2^. In contrast, on 12 May, which was a partly cloudy day, the maximum irradiance was 1321 W/m^2^. For comparison, the irradiance recorded at the same time (12:49:53) on the previous day (11 May) was 950 W/m^2^, indicating that the the corresponding clear-sky irradiance has been enhanced by a factor of 1.4. This comparison illustrates that overirradiance events are less probable on days with clear skies, due to fewer instances of the cloud enhancement effect.

## 5. Conclusions

This work presents the first experiments performed in Belém, Brazil, on monitoring overirradiance events. Accordingly, the irradiance of 1321 W/m^2^ registered on 12 May, corresponds to the first extreme overirradiance event reported at a latitude of 1 °S and an altitude of 7 m a.s.l. These measurements were achieved as a result of modifications made to a previously-reported sensory platform, designed to provide information about overirradiance events on the premises of the Group of Studies and Development in Alternative Energies (GEDAE) of the Federal University of Pará (UFPA). This information is subsequently used in projects concerning the creation of strategies for mitigating the effects of overirradiance in photovoltaic systems.

While these preliminary results are essential for estimating the magnitude of overirradiance events in Belém-PA, further study is required, in trials lasting months to years, to ensure that the reports of overirradiance are representative of the values possible in the region. For instance, higher values of irradiance can be registered in trials conducted in the second half year, because insolation in Belém-PA is at its maximum during this period. The next steps in this work are: To perform tests for a longer duration of time; conduct in-depth studies on the relationship between the cloud enhancement effects and temperature, humidity, and atmospheric pressure; and develop an image acquisition module for capturing pictures of the sky during irradiance events using a cheap, low power fish eye camera.

## Figures and Tables

**Table 1:** Overirradiance events recorded around the world.

References (Year)	Maximum Irradiance (W/m^2^)	Location (Latitude and Altitude above Sea Level)	Instrument of Measurement and Orientation	Resolution of Measurements (s)	Response Time of Instrument, 95% of Final Value (s)
Emck and Ritcher [9] (2008)	1832 W/m^2^	Ecuador (*Andes*), 4 °S, 3400 m.	Thermopile pyranometer (*CM3*) with datalogger (*Kipp & Zonen*), horizontal.	300	≤18
Yordanov et al. [8] (2015)	1600 W/m^2^	Norway (*Grimstad*), 58 °N, 60 m.	Photovoltaic cell *mc-Si* (*Soldata 80spc*) with digital data unit (*Soldata kit*), tilted 39° from horizontal.	10^−2^	≤0.025 *
Almeida et al. [7] (2014)	1590 W/m^2^	Brazil (*São Paulo*), 23 °S, 760 m.	Poly *c-Si* Photovoltaic module (*MSX-10*) with energy analyzer (*Agilent*), horizontal.	1	≤10^−5^
**Present paper**	1321 W/m^2^	Brazil (*Belém-PA*), 1 °S, 7 m.	Mono *c-Si* Photovoltaic Module (SEEED) with the sensory platform (*low-cost*), tilted 10° from horizontal.	1	≤10^−5^
Luoma et al. [4] (2012)	1300 W/m^2^	United States (*San Diego*), 32 °N, 22 m.	Photodiode pyranometer (*LICOR LI-200*) with datalogger (*Kipp & Zonen*), horizontal.	1	≤10^−5^
Piedehierro et al. [3] (2014)	1244 W/m^2^	Spain (*Granada*), 32 °N, 680 m.	Thermopile pyranometer (*CM-11*) with datalogger (*Kipp & Zonen*), horizontal.	60	≤15

^*^ The specified response is the time required for the Soldata 80spc to reach 90% of its final value.

**Table 2:** Financial budget for devices in the platform.

Device	Unit Cost (€) ^1^	Quantity	Total (€)
BMP280	6.67	1	6.67
RTC DS3231	2.85	1	2.85
SD module R/W	2.24	1	2.24
SD card (2 GB)	2.91	1	2.91
INA219	6.04	3	18.12
TP4056	2.21	1	2.21
Li-ion battery (ICR18650, 2200 mAh)	5.06	3	15.2
Photovoltaic module (mono *c-Si*, 0.5 Wp)	2.14	1	2.14
Photovoltaic module (mono *c-Si*, 3 Wp)	4.48	1	4.48
ESP8266 (*NodeMCU*)	8.69	1	8.69
Plastic case (IP67)	3.80	1	3.80
Total (€)			69.31

^1^ Prices in Feb 2018.
